# Supplementary material for: The human glucocorticoid receptor variant rs6190 increases blood cholesterol and promotes atherosclerosis
Source: J Clin Invest. 2025 Jul 1;135(17):e190180. doi: 10.1172/JCI190180 (PMC12404749; doi:10.1172/JCI190180)
Supplement: Unedited blot and gel images [file jci-135-190180-s153.pdf]

WB originals

Figure 2E

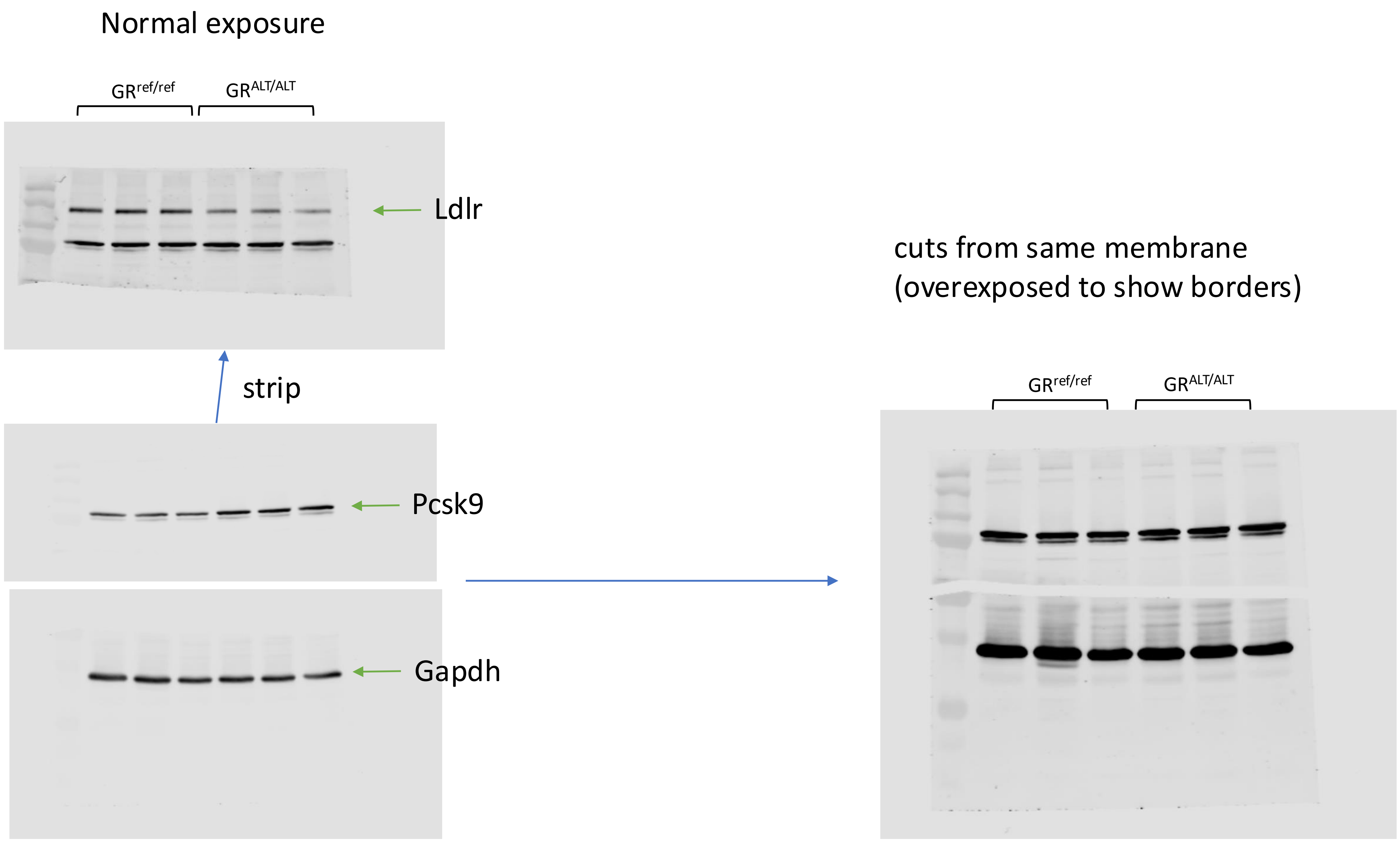

Normal exposure
GR$^{ref/ref}$
GR$^{ALT/ALT}$
Ldlr
strip
Pcsk9
Gapdh
cuts from same membrane
(overexposed to show borders)
GR$^{ref/ref}$
GR$^{ALT/ALT}$

Figure 2E

Normal exposure

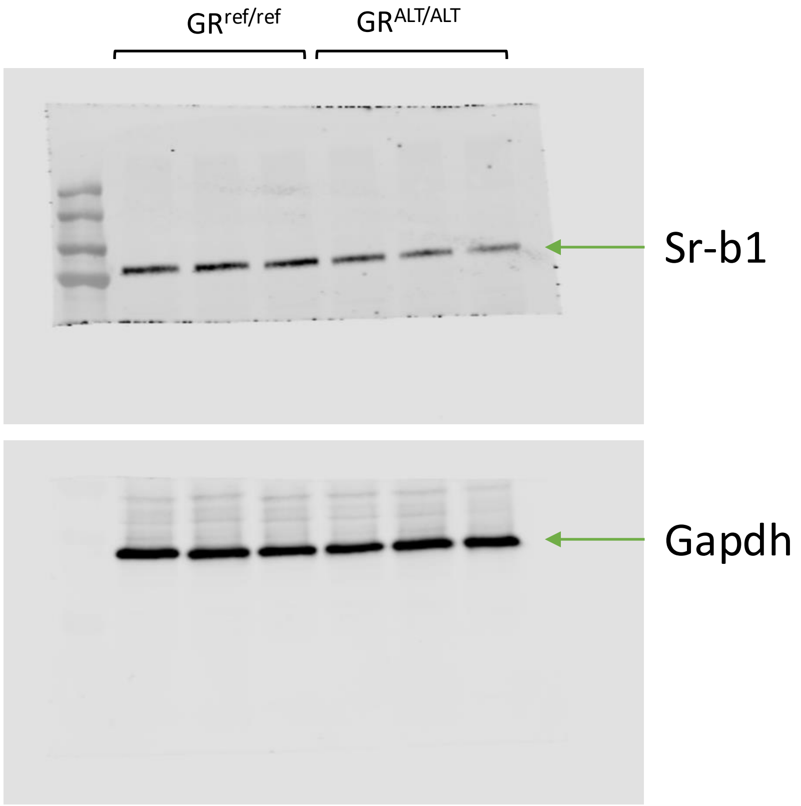

cuts from same membrane
(overexposed to show borders)

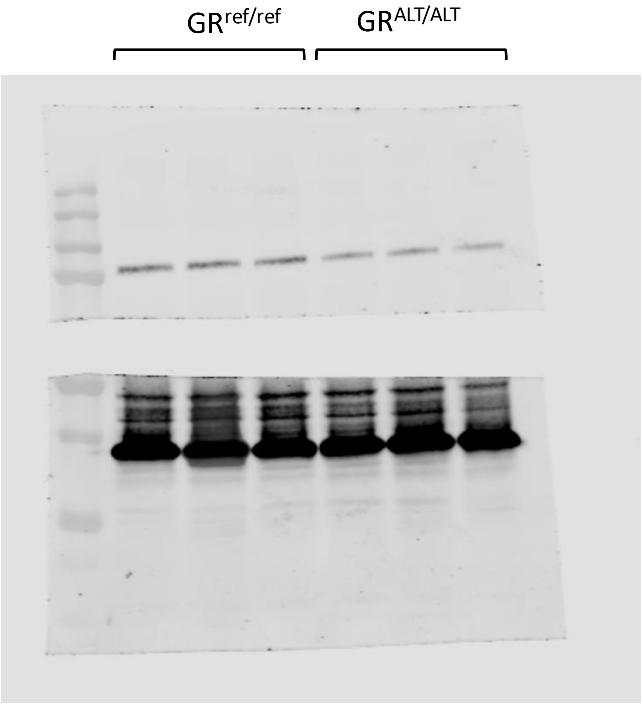

Figure 2E

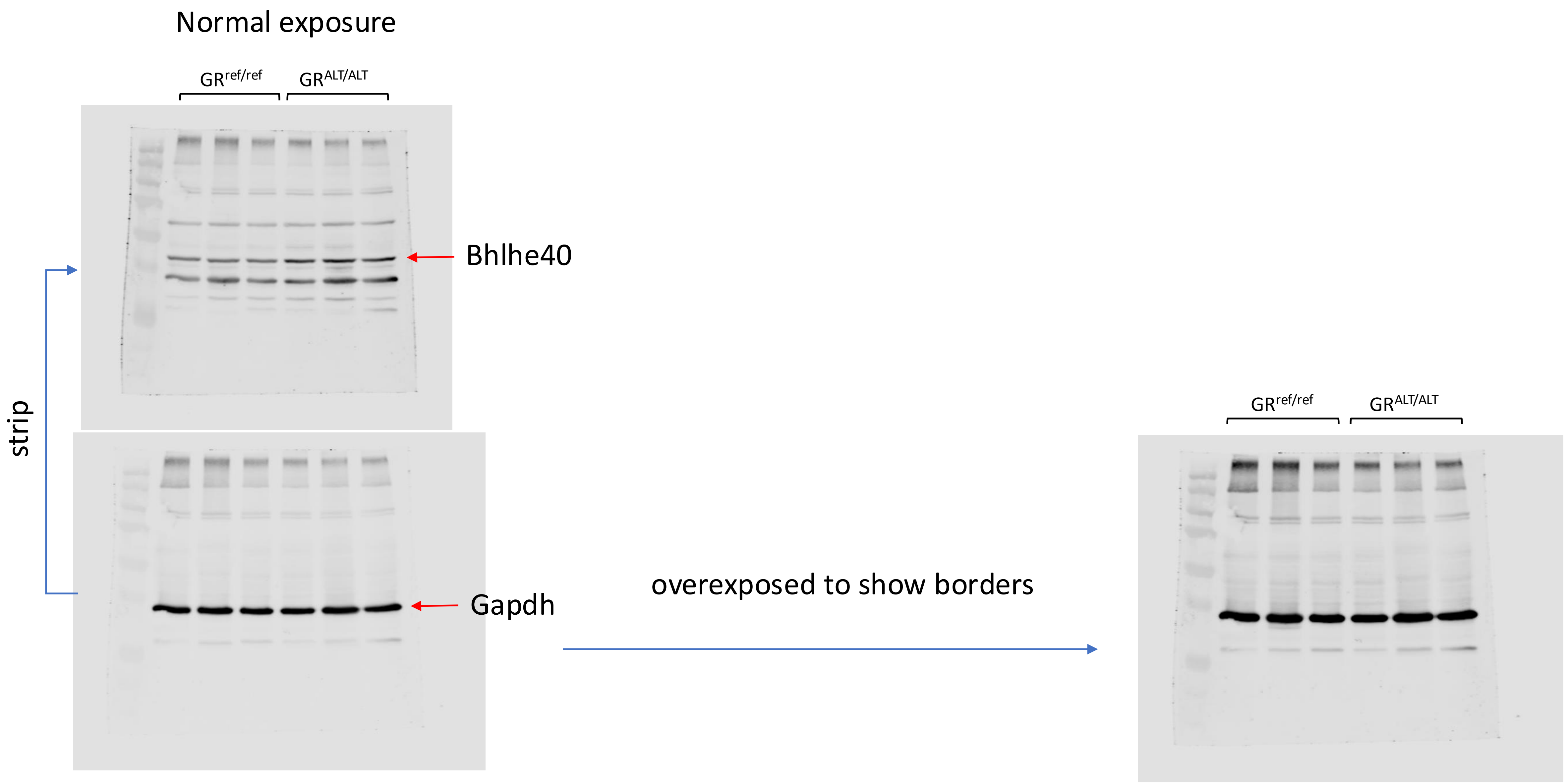
Normal exposure
GR ref/ref
GR ALT/ALT
Bhlhe40
strip
Gapdh
overexposed to show borders
GR ref/ref
GR ALT/ALT

Suppl. Fig. 2D

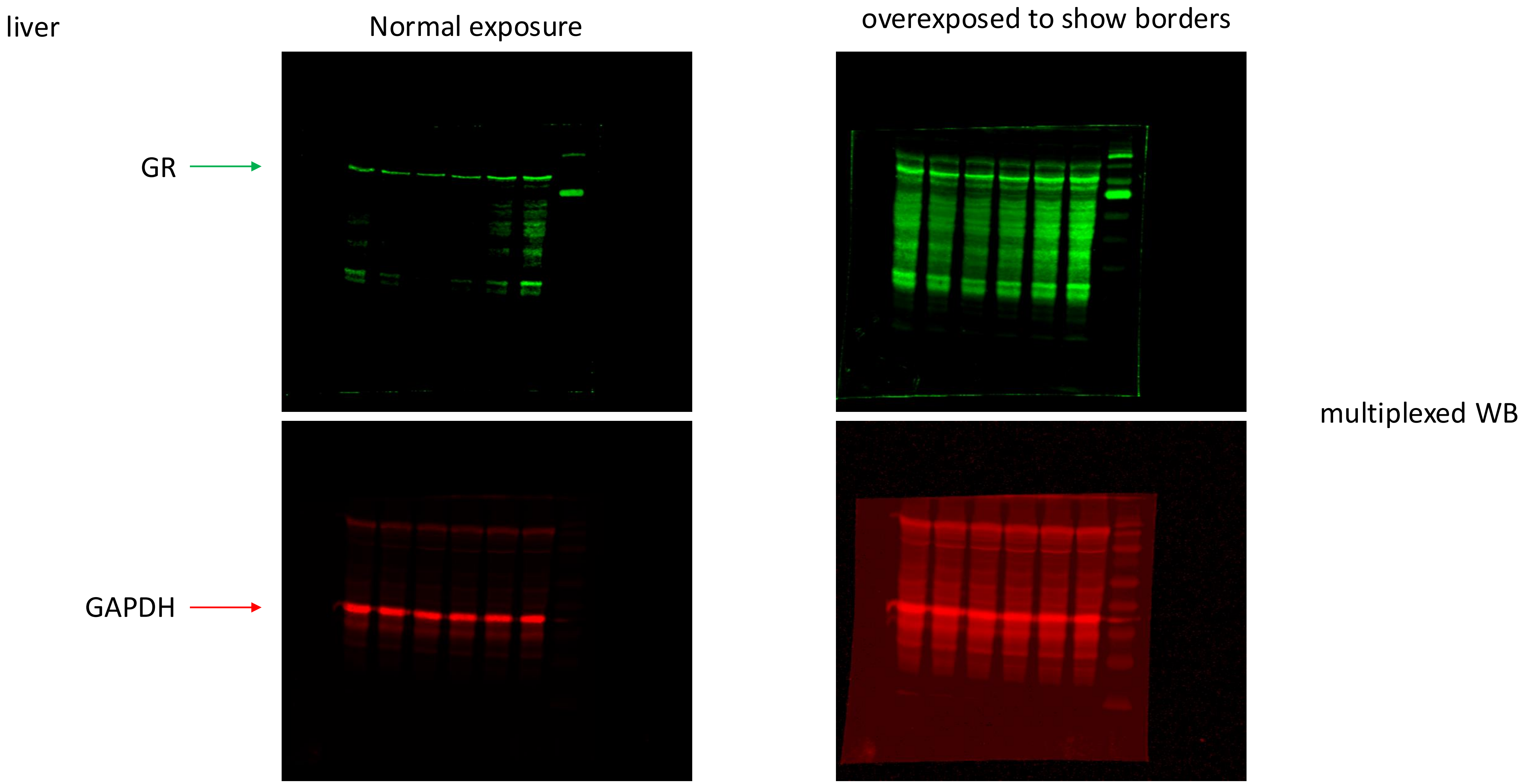
liver
Normal exposure
overexposed to show borders
GR
GAPDH
multiplexed WB

Suppl. Fig. 2D

primary hepatocytes

Normal exposure

overexposed to show borders

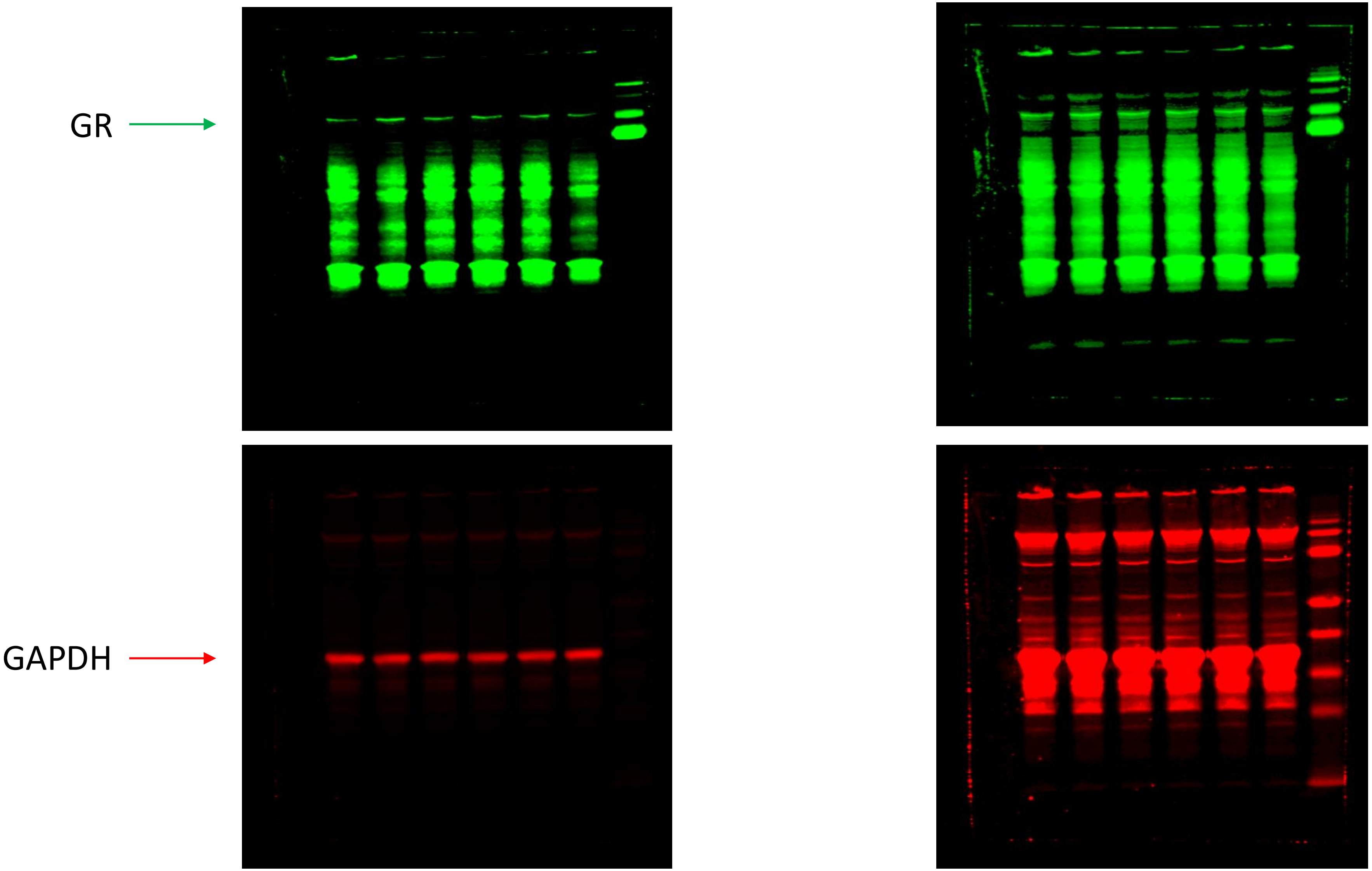

multiplexed WB

Suppl. Fig. 2D

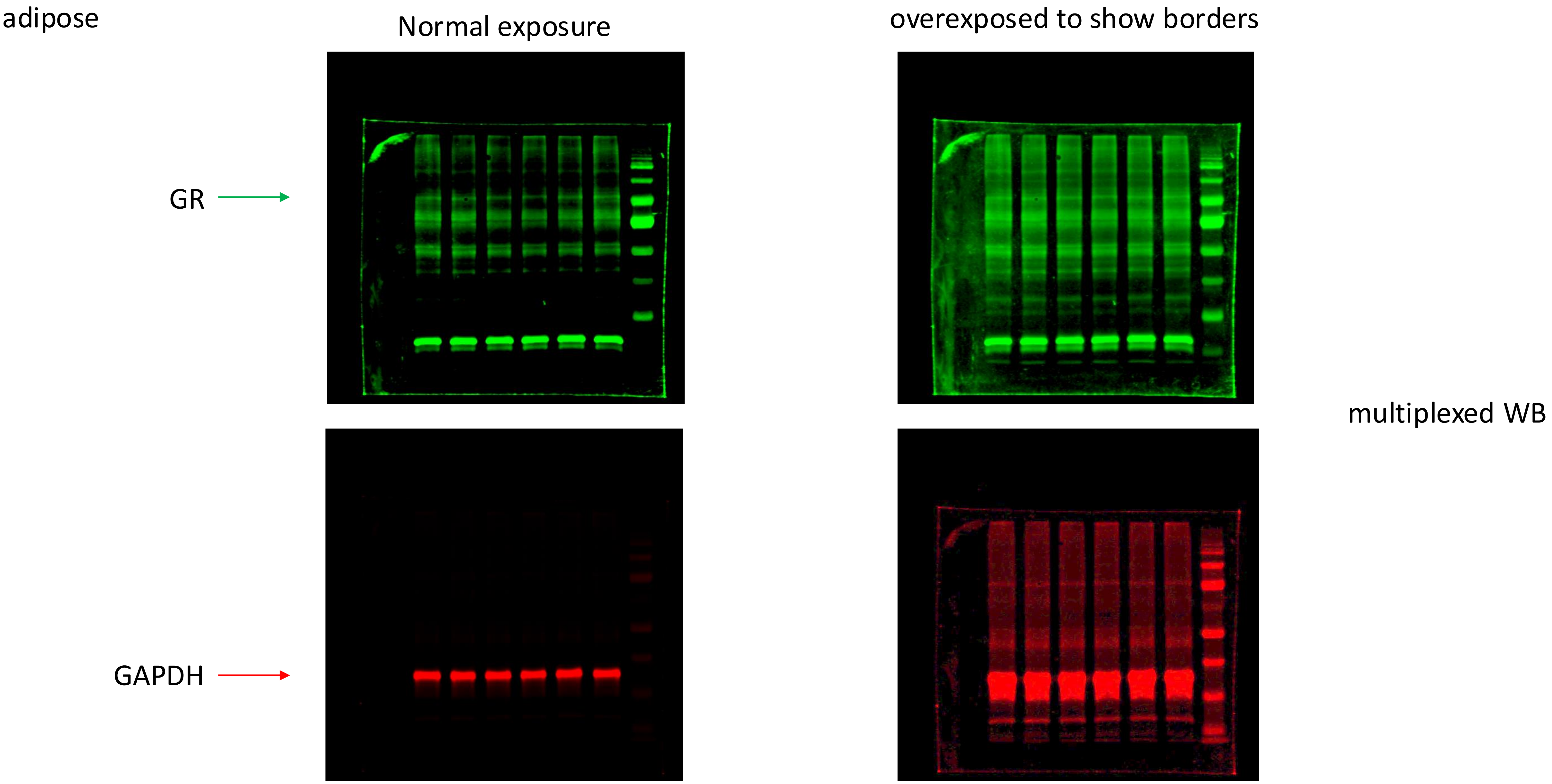
adipose
Normal exposure
overexposed to show borders
GR
GAPDH
multiplexed WB

Suppl. Fig. 2D

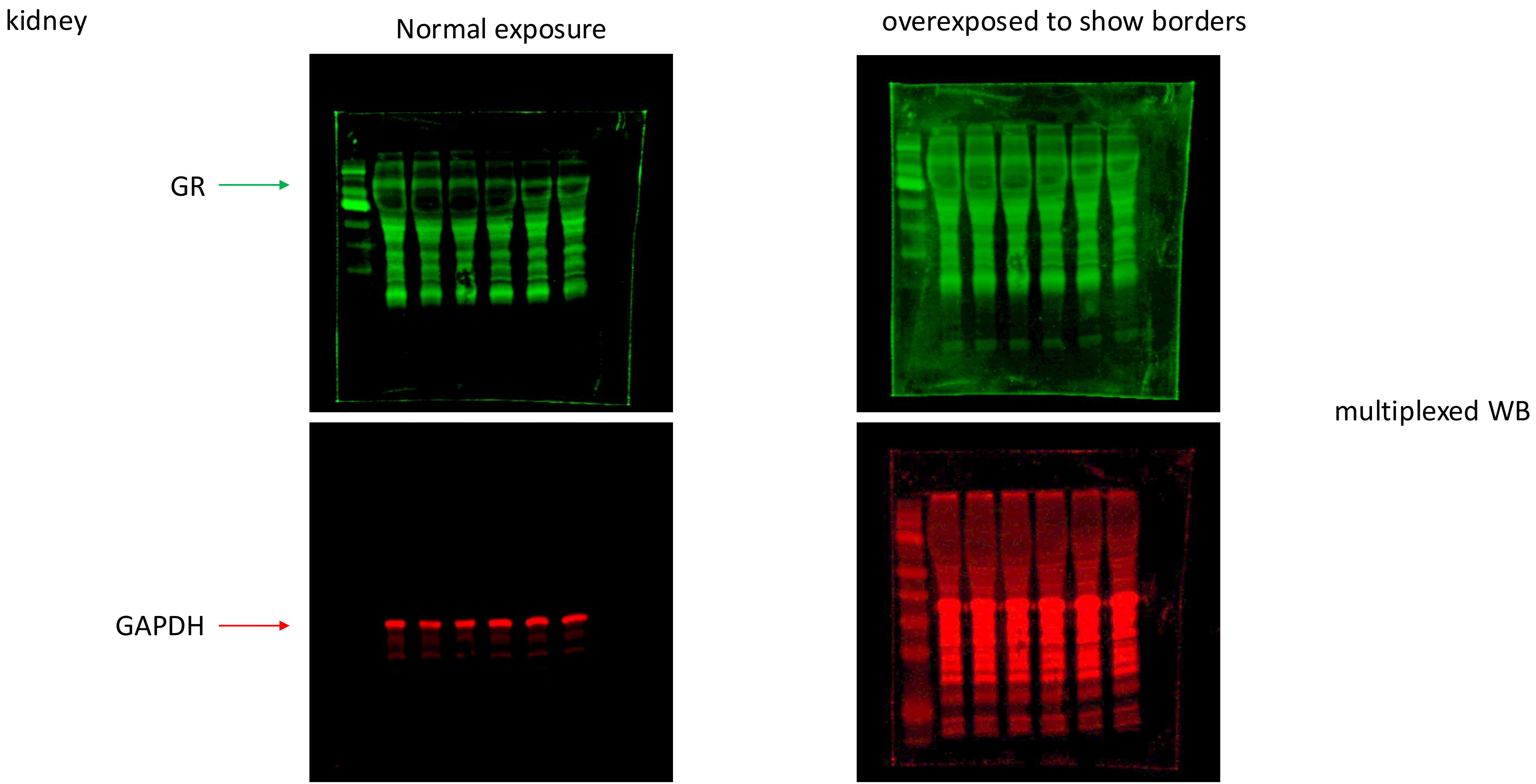
kidney
Normal exposure
overexposed to show borders
GR
GAPDH
multiplexed WB

Suppl. Fig. 3A – 3C GR-Hsp90 CoIP validations

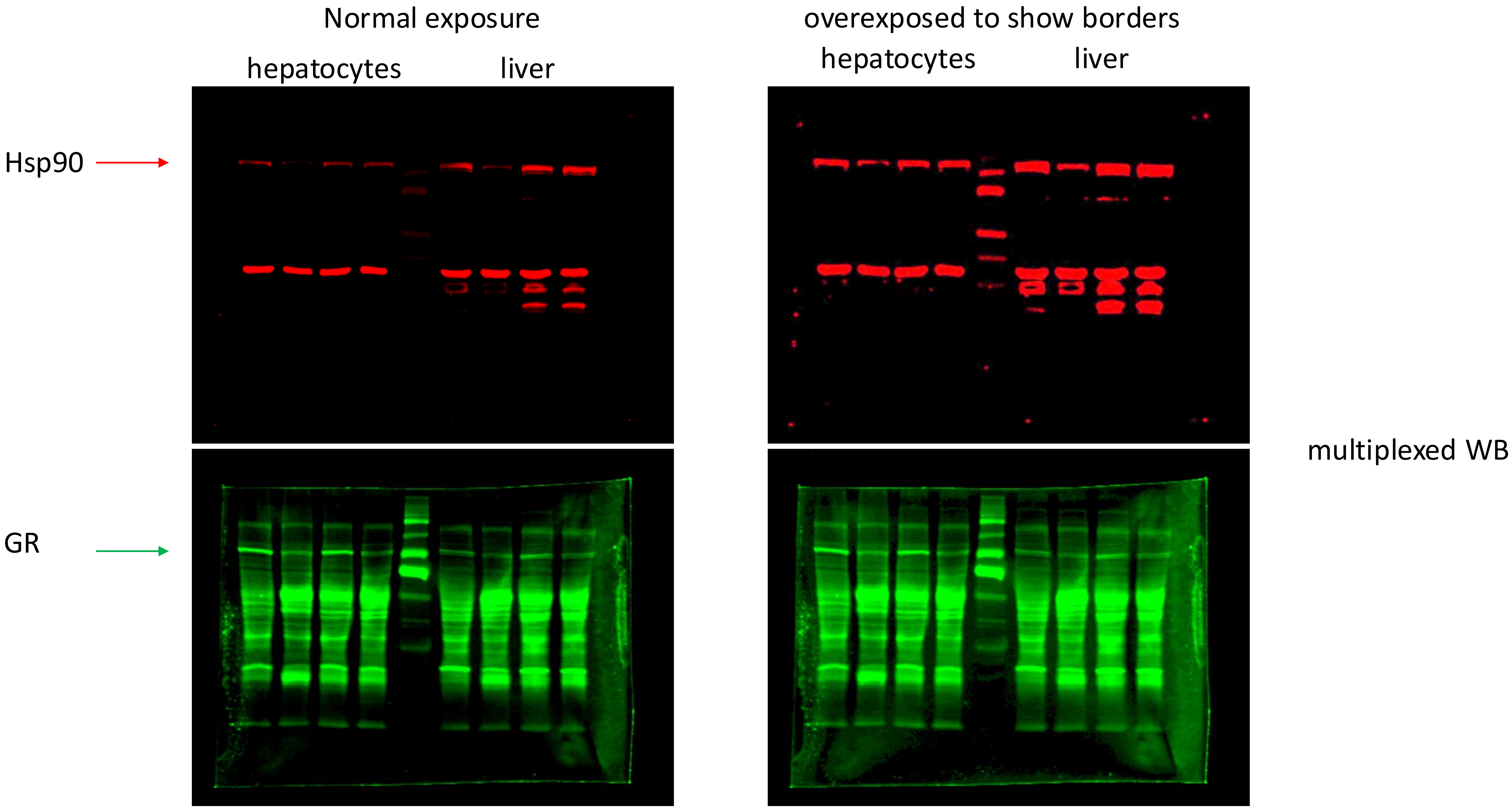

Suppl. Fig. 3A – 3C GR-Hsp90 CoIP validations

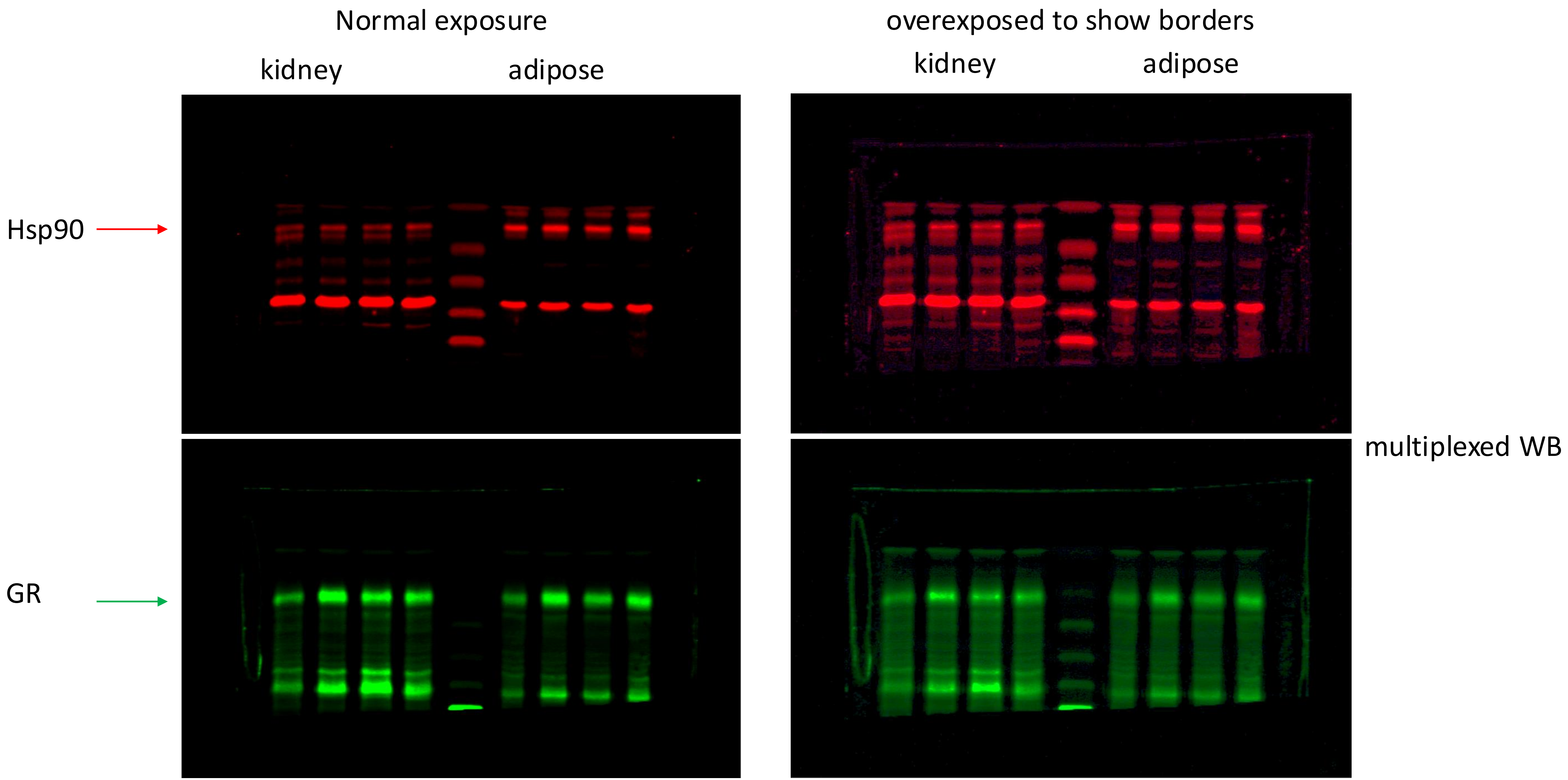

Suppl. Fig. 3B

liver

cuts from same membrane

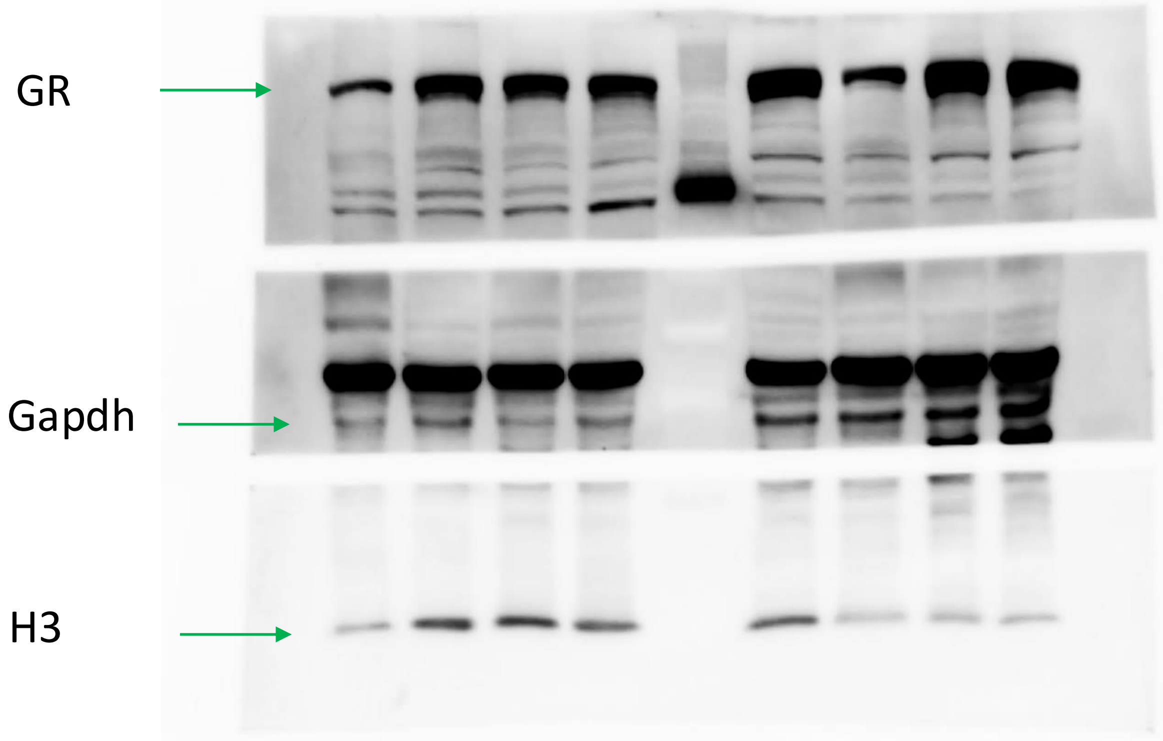

Suppl. Fig. 3B

adipose

cuts from same membrane

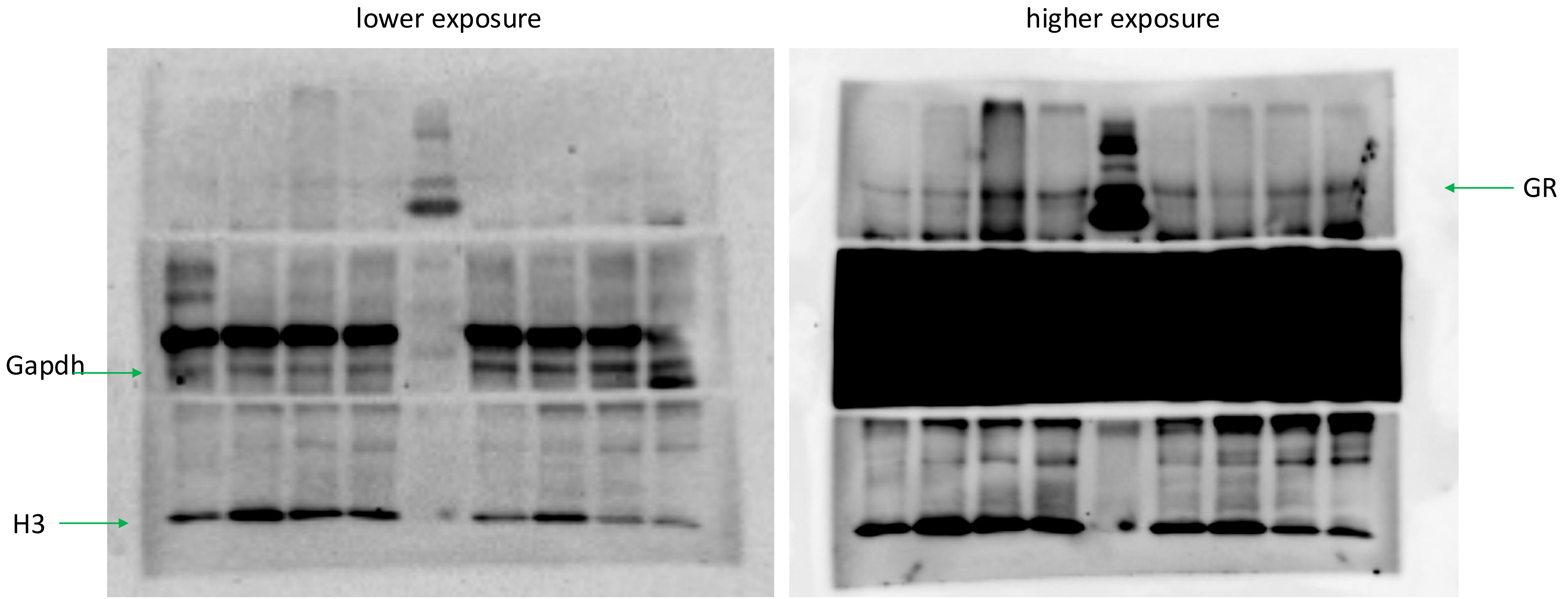

Suppl. Fig. 3C

hepatocytes

cuts from same membrane

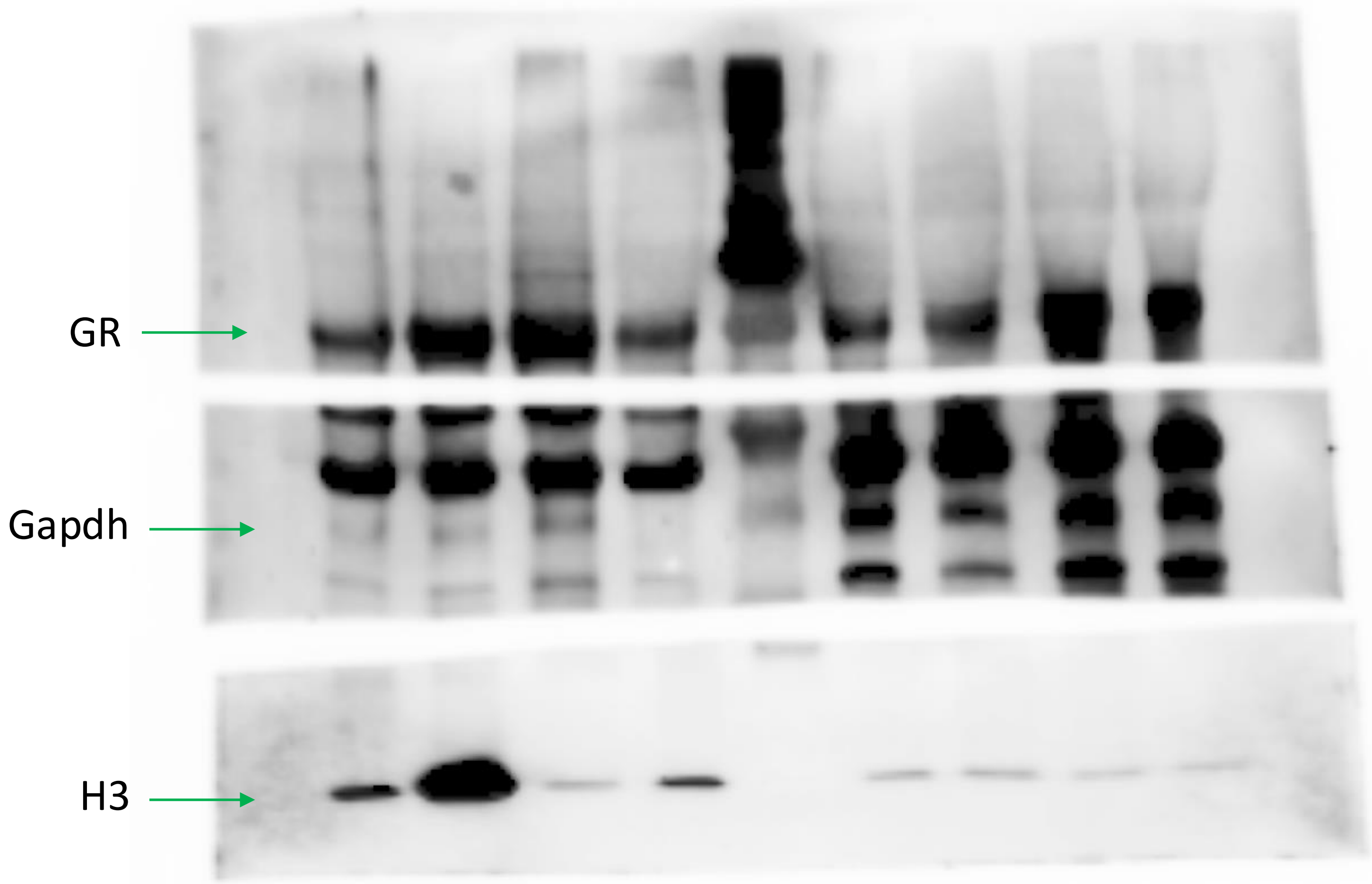

Suppl Fig 3J

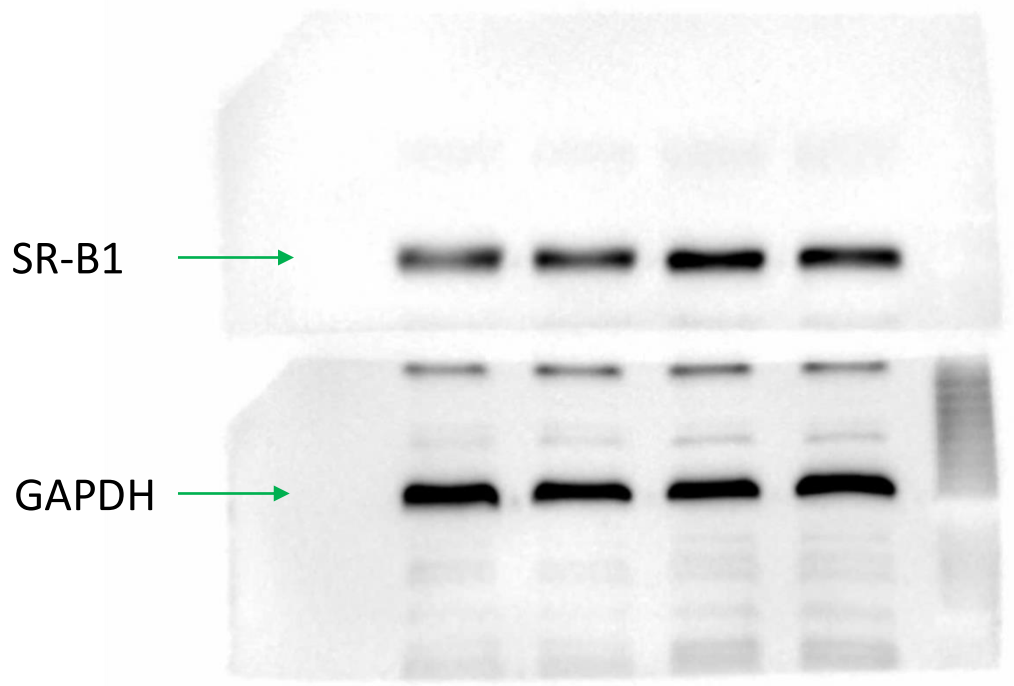

Figure 3D

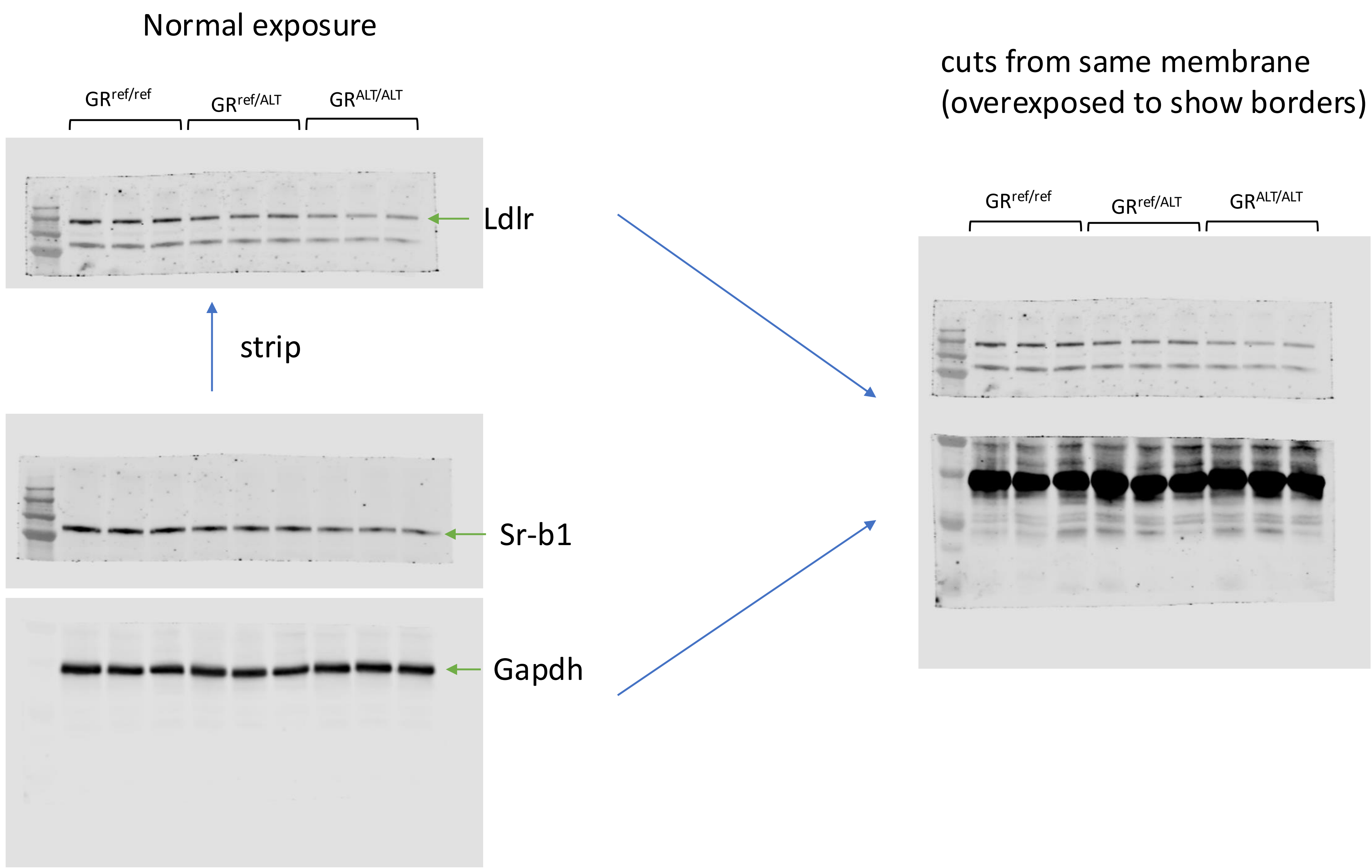

Normal exposure
GRref/ref
GRref/ALT
GRALT/ALT
Ldlr
strip
Sr-b1
Gapdh
cuts from same membrane
(overexposed to show borders)
GRref/ref
GRref/ALT
GRALT/ALT

Fig. 4C

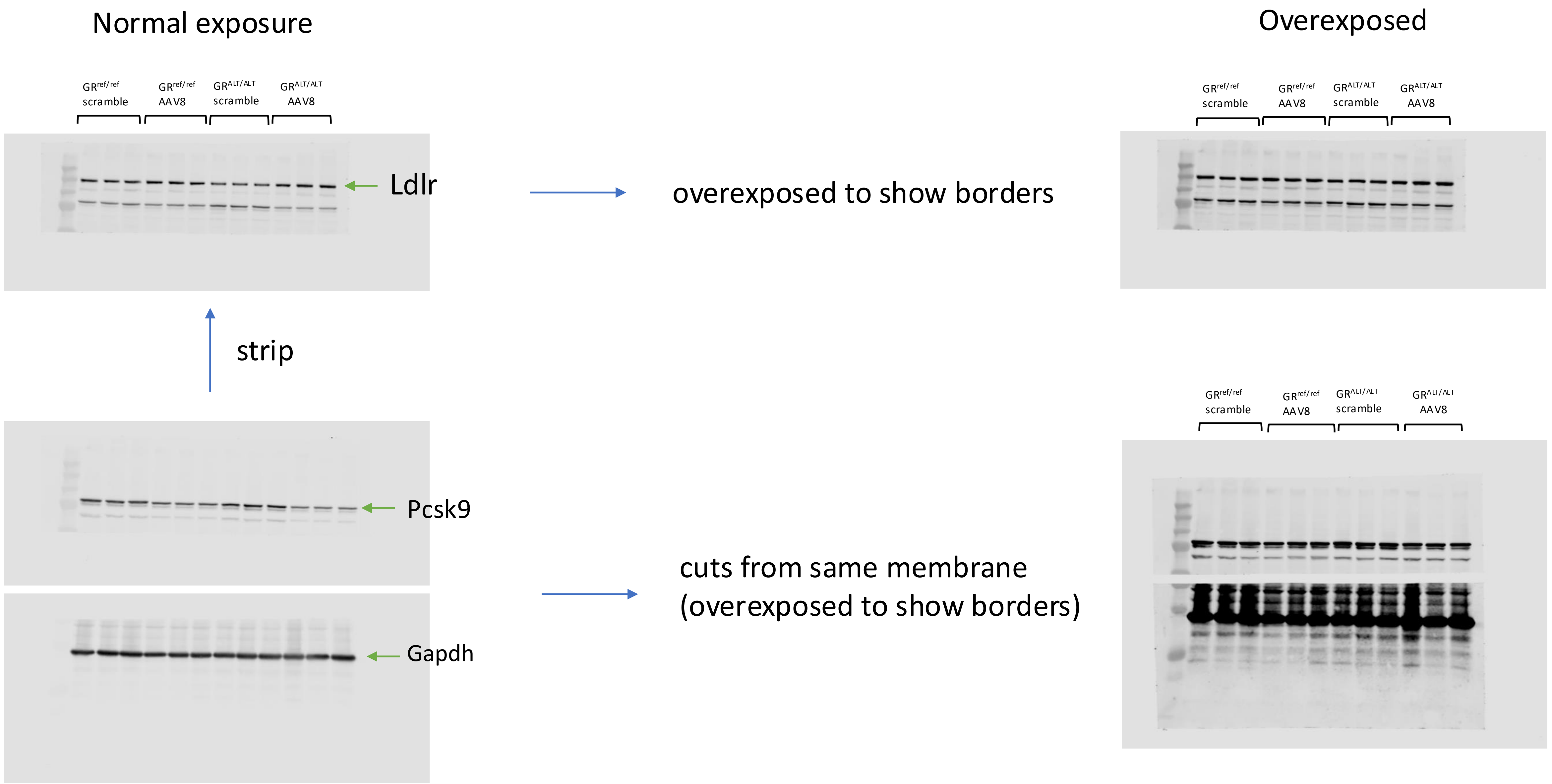
Normal exposure
Overexposed
GR ref/ref scramble
GR ref/ref AAV8
GR ALT/ALT scramble
GR ALT/ALT AAV8
Ldlr
overexposed to show borders
strip
Pcsk9
Gapdh
cuts from same membrane
(overexposed to show borders)

Fig. 4C

Normal exposure

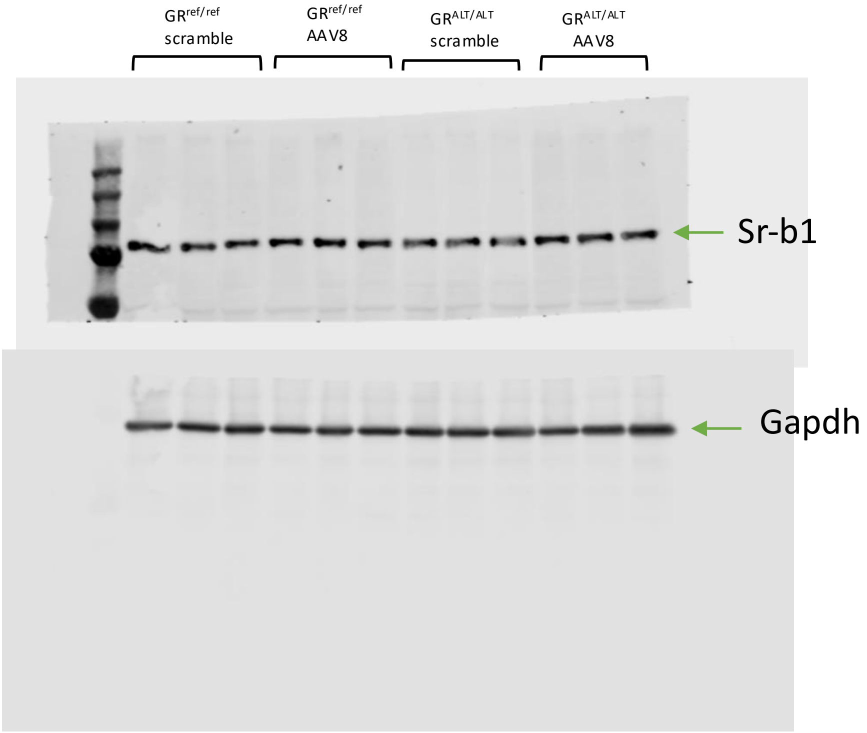

cuts from same membrane (overexposed to show borders)

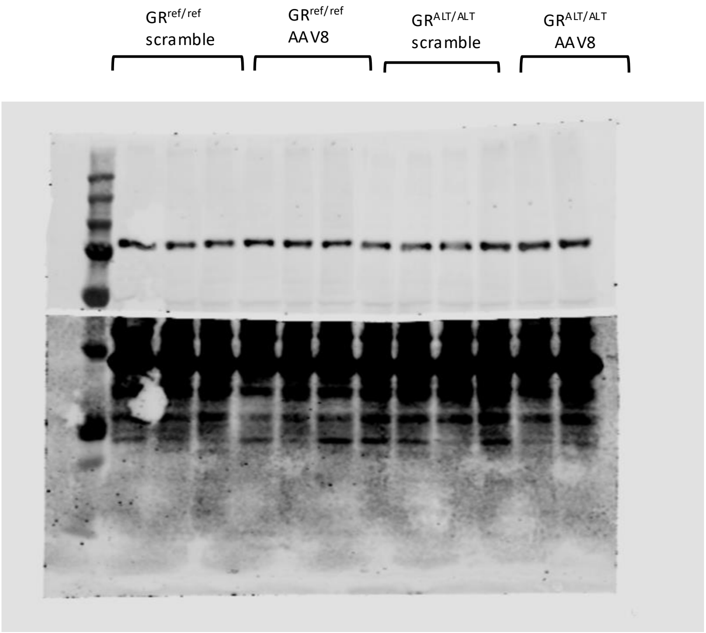

Fig. 4C

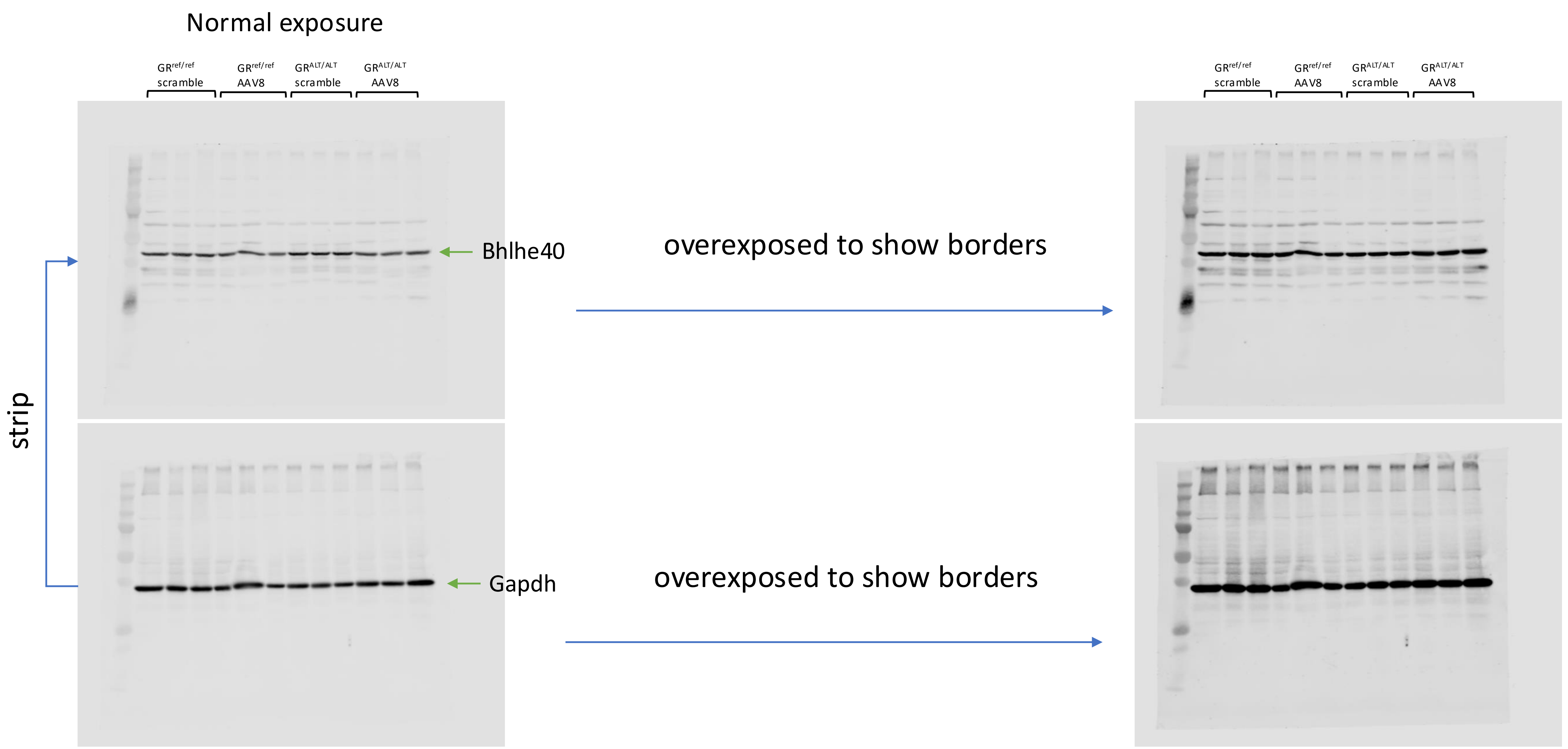
Normal exposure
GRref/ref scramble
GRref/ref AAV8
GRALT/ALT scramble
GRALT/ALT AAV8
Bhlhe40
Gapdh
strip
overexposed to show borders
overexposed to show borders

Fig. 5B

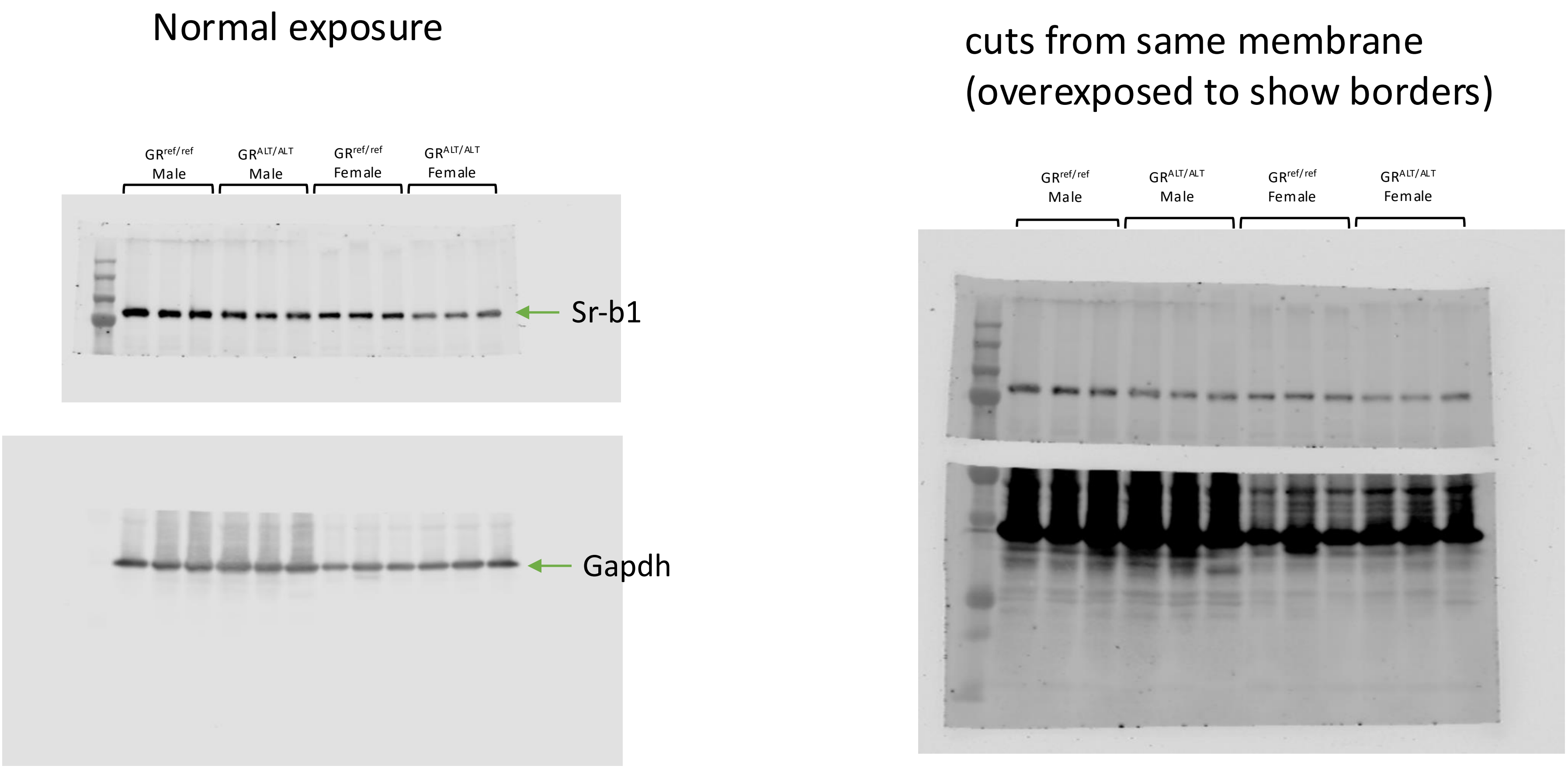
Normal exposure
GR$^{ref/ref}$ Male
GR$^{ALT/ALT}$ Male
GR$^{ref/ref}$ Female
GR$^{ALT/ALT}$ Female
Sr-b1
Gapdh
cuts from same membrane
(overexposed to show borders)
GR$^{ref/ref}$ Male
GR$^{ALT/ALT}$ Male
GR$^{ref/ref}$ Female
GR$^{ALT/ALT}$ Female

Fig. 5B

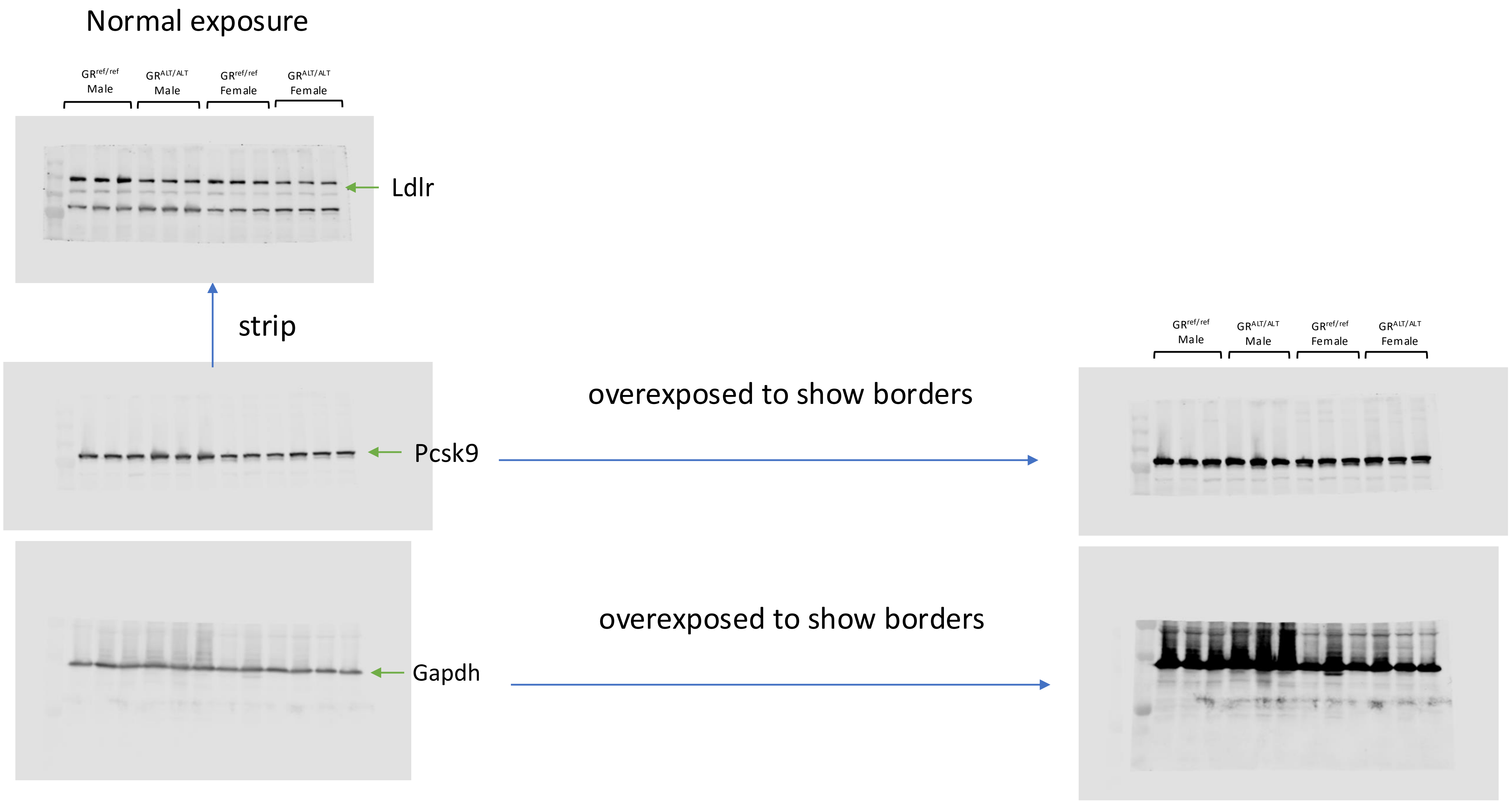
Normal exposure
$GR^{ref/ref}$ Male
$GR^{ALT/ALT}$ Male
$GR^{ref/ref}$ Female
$GR^{ALT/ALT}$ Female
Ldlr
strip
Pcsk9
Gapdh
overexposed to show borders
overexposed to show borders
$GR^{ref/ref}$ Male
$GR^{ALT/ALT}$ Male
$GR^{ref/ref}$ Female
$GR^{ALT/ALT}$ Female

Fig. 5B

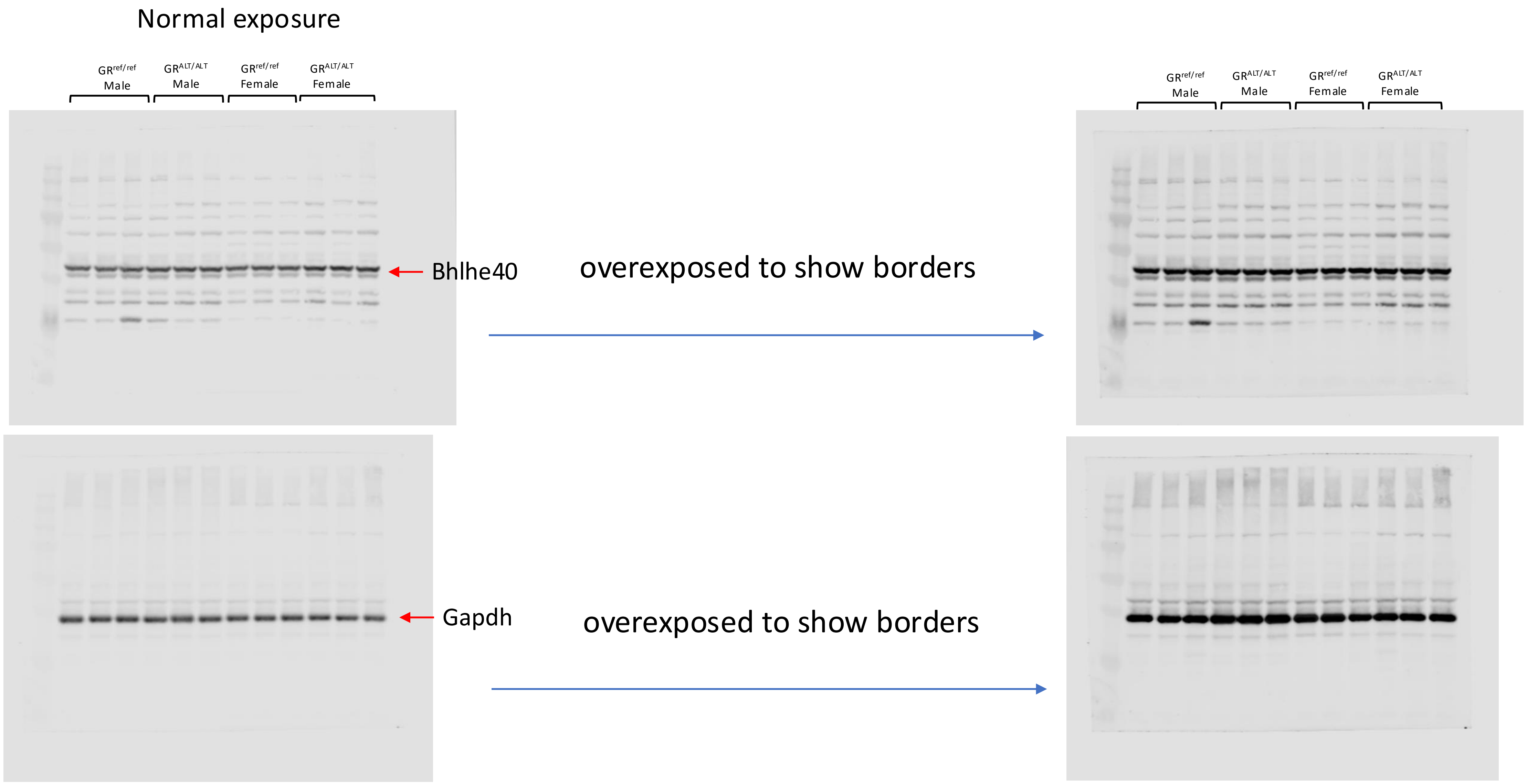

Normal exposure
$GR^{ref/ref}$ Male
$GR^{ALT/ALT}$ Male
$GR^{ref/ref}$ Female
$GR^{ALT/ALT}$ Female
Bhlhe40
overexposed to show borders
$GR^{ref/ref}$ Male
$GR^{ALT/ALT}$ Male
$GR^{ref/ref}$ Female
$GR^{ALT/ALT}$ Female
Gapdh
overexposed to show borders
